# Pathology Reporting in Neuroendocrine Neoplasms of the Digestive System: Everything You Always Wanted to Know but Were Too Afraid to Ask

**DOI:** 10.3389/fendo.2021.680305

**Published:** 2021-04-23

**Authors:** Manuela Albertelli, Federica Grillo, Fabio Lo Calzo, Giulia Puliani, Carmen Rainone, Annamaria Anita Livia Colao, Antongiulio Faggiano, NIKE group, Barbara Altieri

**Affiliations:** ^1^ Endocrinology Unit, Department of Internal Medicine and Medical Specialties (DiMI) and Centre of Excellence for Biomedical Research (CEBR), University of Genova, Genova, Italy; ^2^ IRCCS Ospedale Policlinico San Martino, Genova, Italy; ^3^ Anatomic Pathology Unit, Department of Surgical Sciences and Integrated Diagnostics (DISC), University of Genova, Genova, Italy; ^4^ Department of Clinical Medicine and Surgery, Division of Endocrinology, Federico II University, Naples, Italy; ^5^ Internal Medicine Unit, Frangipane Hospital, Ariano Irpino, Italy; ^6^ Department of Experimental Medicine, Sapienza University of Rome, Roma, Italy; ^7^ Oncological Endocrinology Unit, Regina Elena National Cancer Institute, Roma, Italy; ^8^ UNESCO Chair for Health Education and Sustainable Development, Federico II University, Naples, Italy; ^9^ Endocrinology Unit, Department of Clinical and Molecular Medicine, Sant’Andrea Hospital, Sapienza University of Rome, Roma, Italy

**Keywords:** neuroendocrine neoplasms (NENs), neuroendocrine classification, immunohistochemistry, pathology, morphology, grade, Ki67

## Abstract

During the 5th NIKE (Neuroendocrine tumors Innovation in Knowledge and Education) meeting, held in Naples, Italy, in May 2019, discussions centered on the understanding of pathology reports of gastroenetropancreactic neuroendocrine neoplasms. In particular, the main problem concerned the difficulty that clinicians experience in extrapolating relevant information from neuroendocrine tumor pathology reports. During the meeting, participants were asked to identify and rate issues which they have encountered, for which the input of an expert pathologist would have been appreciated. This article is a collection of the most rated questions and relative answers, focusing on three main topics: 1) morphology and classification; 2) Ki67 and grading; 3) immunohistochemistry. Patient management should be based on multidisciplinary decisions, taking into account clinical and pathology-related features with clear comprehension between all health care professionals. Indeed, pathologists require clinical details and laboratory findings when relevant, while clinicians require concise and standardized reports. In keeping with this last statement, the minimum requirements in pathology datasets are provided in this paper and should be a baseline for all neuroendocrine tumor professionals.

## Introduction

During the 5th NIKE (Neuroendocrine tumors Innovation in Knowledge and Education) meeting, held in Naples, Italy, in May 2019, discussions centered on the understanding of pathology reports in gastroenetropancreactic (GEP) neuroendocrine neoplasms (NENs). In particular, the main problem concerned the difficulty clinicians (be they experts or not) have, in extrapolating relevant information from neuroendocrine tumor pathology reports. As the famous publication entitled “Clinicians are from Mars and pathologists are from Venus” (1), perfectly summed up, this is not a new issue. During the meeting, participants were asked to identify issues which they have encountered, for which the input of an expert pathologist would have been appreciated. This article is a collection of the most rated questions, focusing on three main topics: 1) morphology and classification; 2) Ki67 and grading; 3) immunohistochemistry.

## Methods

A series of questions on various aspects of pathology were proposed to a panel of 36 experts in the field of GEP-NENs (including endocrinologists, pathologists, oncologists, gastroenterologists, surgeons, radiologists and laboratory clinicians; see acknowledgment section). All questions are summarized in **Table 1S**, along with the rate of votes obtained during the poll (participants could select a total of 8 questions), while the top scored questions are answered below.

## Morphology and Classification: Questions and Answers

### Question 1: What Classification System for Neuroendocrine Neoplasms of the Digestive System Should I Be Expecting in a Pathology Report and Does the Category NET G3 Really Exist?

Classification systems for NENs have varied over time, each one emphasizing different aspects including function, morphology, site, size and extension of primary tumor, presence of metastases.

**Figure 1:**
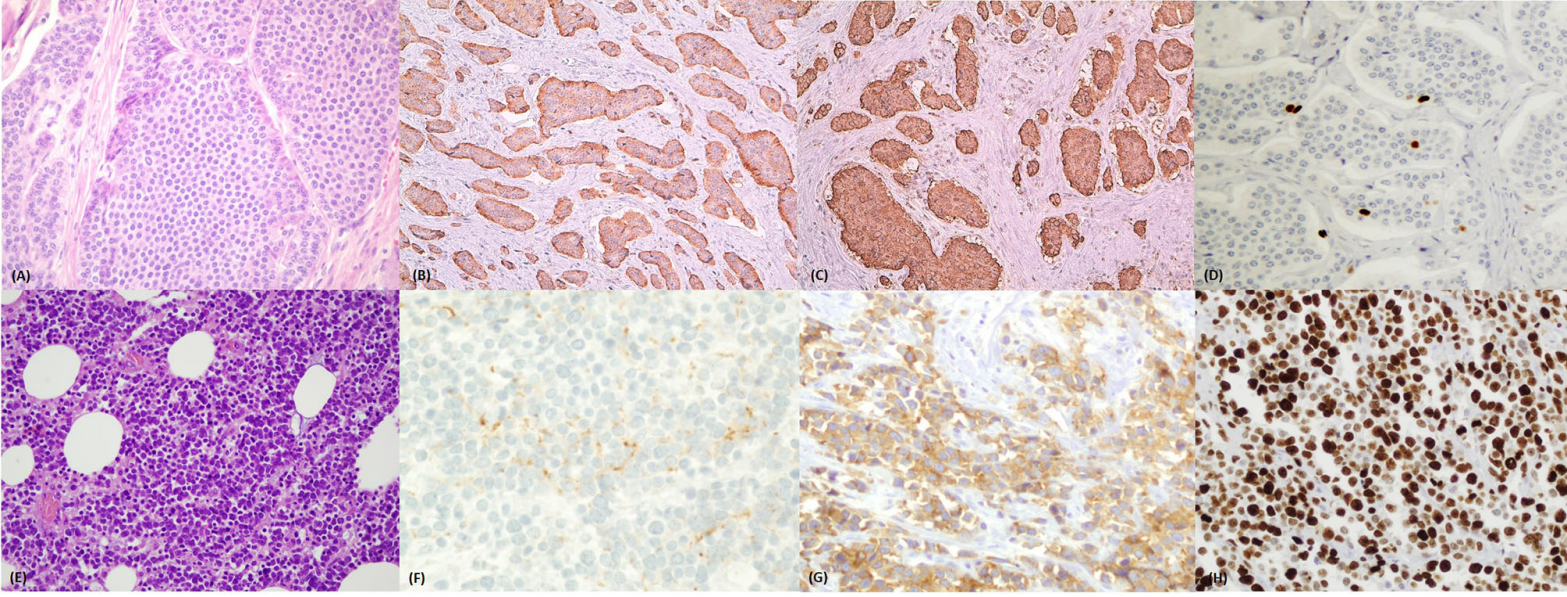
**(A–D)** Well differentiated neuroendocrine tumor of the ileum. **(A)** Haematoxylin and eosin stained section (magnification 40x) of a well differentiated ileal neuroendocrine tumor with organoid insular architecture and monomorphic cells with ample eosinophilic cytoplasm and uniform nuclei. **(B)** Chromogranin A positivity and **(C)** synaptophysin positivity by immunohistochemistry. **(D)** Ki67 immunostaining showing rare positive nuclei (stained brown) with <3% proliferation ratio – grade 1. **(E–H)** Poorly differentiated neuroendocrine carcinoma of the colon. **(E)** Haematoxylin and eosin stained section (magnification 40x) of a poorly differentiated neuroendocrine carcinoma showing solid structure and small/moderate atypical cells with scanty cytoplasm and hyperchromatic nuclei. **(F)** Focal dot like positivity for Chromogranin A but diffuse, cytoplasmic expression of synaptophysin **(G)**. **(H)** Ki67 immunostaining showing diffusely positive nuclei (stained brown) with 90% proliferation ratio – grade 3.

With the 2010 GEP NEN WHO classification (2), a morphology and proliferation-based classification system was introduced. It focused on the morphologic distinction between well differentiated (WD neuroendocrine tumors - NET) and poorly differentiated (PD neuroendocrine carcinomas - NEC) neoplasms, as already suggested in the WHO 2000 classification (3). WD-NETs are composed of uniform neoplastic cells, with organoid, trabecular or ribbon-like architecture, round/oval nuclei with “salt and pepper” chromatin and with low nuclear-cytoplasmic ratio. They present secretory granules responsible for intense and diffuse staining for general neuroendocrine markers (synaptophysin and chromogranin) (**Figures 1A–D**). Nucleoli are inconspicuous and little or no atypia is seen. Mitoses are rare/uncommon and necrosis is also generally absent. PD-NECs, are either of large cell or small cell type (or mixed), with pleomorphic and atypical nuclei, solid growth pattern and abundant non-ischemic necrosis, arranged to form either ‘‘map-like” or ‘‘spotty” necrosis. Mitoses are plentiful and often atypical (4) and proliferation index is extremely high (**Figures 1E–H**).

The second aspect of the 2010 WHO classification, which has now become paramount for patient management, is grade, based on mitotic index and/or Ki67 index (see question 3). Initially, categories comprised G1 and G2 WD-NETs and G3 PD-NECs. Some G3 neoplasms were, however, found to be morphologically well differentiated (perhaps with focal areas of greater atypia) but with proliferation indexes greater than 20% [around 45% (5), usually no higher than 50-60%] (6, 7). Subsequent studies have shown that WD G3 neoplasms are a separate category showing better survival compared to PD G3 carcinomas (but worse compared to G2 NET) (8), somatostatin receptor positivity (9), Gallium-PET positivity (with associated possible FGD-PET positivity) and, at the molecular level, mutation profiles similar to WD G1/G2 tumors (10). The landmark study by Sorbye et al (11) reported differences in response to chemotherapy between G3 NENs with Ki67 < 55% compared to > 55%; this study unfortunately failed to review the morphology of the accrued cases.

A revised common classification system of GEP-NENs was therefore proposed for pancreatic NENs in 2017 (12) and extended to all digestive NENs in 2019 (13).

With regards to stage, the 2017 edition of the UICC/AJCC staging manual has specified site specific TNM systems for well differentiated GEP NETs including gastric, duodenal/ampullary, jejunal/ileal, appendiceal, colonic/rectal and pancreatic NETs. The use of this updated system should be standard in all pathology reports.

### Question 2: How Should I Interpret a Pathology Report Showing a Digestive System Mixed Neuroendocrine Non-Neuroendocrine Carcinoma?

Mixed neuroendocrine/non-neuroendocrine neoplasms have been described in all organs of the digestive system, with highest frequency in the colon and a diagnostic requirement is that both components be at least 30% of the lesion (though this cut off is arbitrary and not evidence-based). The WHO 2010 classification recommended the term mixed adeno-neuroendocrine carcinoma (MANEC) (2) for such tumors however, this term, does not adequately cover the heterogeneity of possible combinations of neuroendocrine (WD or PD) and non-neuroendocrine (adenocarcinoma, squamous cell carcinoma or adenoma for example) phenotypes.

For this reason, the 2017-2019 WHO classifications changed the term to mixed neuroendocrine and non-neuroendocrine neoplasms (MiNEN). These neoplasms can be stratified into different prognostic categories according to the grade of malignancy of each component: low-grade MiNENs (adenoma and a WD-NET, called MANETs (14); high-grade MiNENs, (PD-NEC with adenocarcinoma, called MANEC or squamous carcinoma in the esophagus or anal canal); intermediate grade neoplasms, composed of adenocarcinoma and NET (15, 16).

In general, the most aggressive cell population drives clinical behavior and this should be considered for therapeutic strategy (17). Recent studies on digestive system MiNENs have shown that prognosis is driven mostly by the NEC component when present, and often, it is this component which metastasizes (18). The Ki67 proliferative index of the neuroendocrine component appears to be the key prognostic factor with differences in survival if Ki67 is above 55% (17). Similarly, Ki67 of 55% seems to be important in composite lung large cell neuroendocrine carcinomas also (19).

With regards to origin, a few studies have demonstrated that both the high-grade NEC component and the non-neuroendocrine component probably derive from the same precursors as they show similar mutation profiles (20).

## Grading and Ki67 Evaluation: Questions and Answers

### Question 3: Why Is Grade so Important in NENs and How Reliable Is Ki67 Evaluation on Cytological or Small Tissue Samples?

Grade represents a major prognostic factor (21, 22) and is evaluated on the basis of mitotic index and proliferative index (Ki67 immunostaining) evaluated on sections of tumor. Ki67 is a nuclear protein expressed in the active phases of the cell cycle (G1/S1/G2/M phases) and its function is as a biological surfactant to disperse mitotic chromosomes (23). Discordance between grade assessed by mitotic counting or by Ki67 index is often seen (about 30% of cases), and grade is usually higher when assessed by Ki67 (24, 25). In WHO 2017-2019 (12, 13), grade cut offs have been slightly modified between G1 and G2 so that no grey zone (between 2 and 3%) exists; the distinction between G1 and G2 tumors is now <3% Ki67 index and <2 mitosis/10 high power fields (HPF).

The suggested number of cells in hot spots of expression which should be counted has changed over the years, from 2000 cells in the WHO 2010 to 400-500 cells in the WHO 2017-2019. Furthermore, methods of evaluation of Ki67 have come under scrutiny in recent years as not all methods are equally reliable (26). ‘Eye-ball’ estimation has proved to be unreliable while optimal methods include automated counting by image analyser, manual eye-counting and manual count of camera-captured image. The accuracy and reproducibility of these methods vary in different studies (27, 28).

Besides technical aspects, other possible limitations of Ki67 index assessment derive from the small quantity of tissue available, such as small biopsies (29, 30) and, even more so, in case of cytologic samples. Several studies have focused on the comparison of grading evaluation using endoscopic ultrasound-guided fine needle aspiration and surgical pathology in pancreatic NEN, identifying the correct identification of grade G2 NET as the principal limitation of cytology with both over and undegrading of lesions (31–34). Overall, agreement between cytology and definitive histologic examination was extremely variable in all studies ranging from as low as 34% (31) to close to 100% (35). While it is true that cytology may not be able to accurately predict Ki67 proliferation index in the intermediate range (distinction between G1 from G2 WD-NETs), it is reliable in identifying very proliferative tumors (36) and clinicians should be aware of this.

### Question 4: Is There Intra/Intertumoral Heterogeneity in Grade and Can Grade Change Between Sites and Over Time?

With regards to intratumoral heterogeneity of grade in NENs, this can be seen (up to 77% of patients in a study in small bowel NENs (37)) and may be related to multifocality and size, when primary tumor > 1 cm, making the staining of Ki67 sufficient only in the largest lesion (38).

When considering change in grade, this has been shown to occur between the primary and metastatic sites and between synchronous/metachronous metastases (39). The first published study on this topic identified 49 patients with metastatic GEP-NEN, showing a discrepancy in grade between sites in 39% of cases, especially in distant compared to locoregional metastases (39). Further studies have demonstrated an overall discordance rate between primary and metastatic tumour of between 1/3-1/2, both with regards to increase (including from G1 to G3) and decrease in grade from primary to metastatic sites (40). Importantly, increased grade in metastatic sites is associated with lower progression free survival and overall survival (41–43).

In conclusion, it is very important for the clinician to be aware of the possibility of change of grade between sites and over time and it may become useful to re-evaluate grade on a new biopsy.

## Immunohistochemistry: Questions and Answers

### Question 5: Is it Necessary to Evaluate SSRs on Neoplastic Cells by Immunohistochemistry?

Five somatostatin receptor (SSTR) subtypes have been identified; moreover, two forms of the SSTR2, A and B, are transcripted by alternative splicing, with SSTR2A being the most highly expressed (44). SSTR2 and SSTR5 are the most expressed subtypes and their expression on the membrane of neoplastic cells is the rationale for the use of somatostatin analogues (SSA) and peptide receptor radionuclide therapy in WD-NENs (45, 46). In most cases, functional imaging with 68-Ga-DOTATOC/DOTANOC/DOTATATE PET CT permits the *in vivo* evaluation of receptor expression (47); as an alternative, the presence of SSTR can be demonstrated by immunohistochemistry. SSTR2A monoclonal antibody has shown high sensitivity/specificity and can be used in formalin-fixed and paraffin-embedded tissues. To standardize the interpretation of immunostaining, Volante et al. proposed a score considering the subcellular pattern and the extension of positive neoplastic cell population (48) with demonstrated high interlaboratory and interobserver SSTR2A immunostaining agreement (49).

Clinicians should be aware of the availability of SSTR2 receptor evaluation in those patients who have not undergone pre-operatory nuclear imaging when, for example, the diagnosis of NEN is made after surgery as recommended by ENETS (36).

SSTR2A expression has been shown to be higher in low-grade NENs and decreased in high grade lesions, both in digestive (50) and in lung (51) neoplasms. Studies have proposed a correlation between the downregulation of SSTR2 expression and NEN growth and progression (52) as well as differences in expression in metastatic sites compared to primary (53). SSTR2A expression may also be correlated with prognosis [WD-NETs with high expression of SSTR2 are associated with longer overall survival (54–56)].

While several studies have evaluated the expression of all SSTR subtypes in NEN (57, 58), this profiling is not part of the routine immunohistochemical evaluation. Notwithstanding this, two aspects seem very promising for future applications: the expression of SSTR5, for predicting the additional value of new SSA pasireotide (59) and the identification of the truncated variant of SSTR5 which seems associated with worse prognosis and low response to SSA (60).

### Question 6: How Sensitive/Specific Are Site of Origin Markers (TTF1, CDX2, PAX8, ISL1, PDX1)?

A frequent clinical setting (between 9-19% of NENs) is a patient with multiple liver metastases which show WD-NET and for which the clinician requires, not only a diagnosis of histotype and grade, but an indication of origin as well (61).

Determining the origin of the tumor by histologic features alone is often impossible. The typical neuroendocrine markers used in clinical practice, chromogranin and synaptophysin, do not indicate a specific primary, therefore, further immunohistochemical testing may be required to help pathologists identify primary site. Only in WD-NETs are transcription factors useful and these may be differentially expressed in the bowel (CDX2), lung (TTF1) or pancreas (PAX8, ISL1, PDX1). PD-NECs do not express transcription factors with reliability and these should not be used to identify origin (e.g. TTF1 is often expressed in PD-NECs of any site, including the digestive system).

CDX2 is a nuclear homeobox transcription factor responsible for development of all (neuroendocrine and non-neuroendocrine) intestinal epithelial cells. High prevalence of CDX2 expression was found in ileal (86%) and colonic (75%) NETs while no expression was found in NETs of gastric origin, lung, skin, ovary and thymus (62). CDX2 expression has however been reported in a low percentage of pancreatic NETs (pNETs) (15-26%) (62, 63). Worthy of note is that CDX2 has been shown to be expressed in up to 98% of appendiceal and rectal NETs which originate from enterochromaffin cells (serotonin producing) but not from L-cell NETs (which may be found at both sites) (64).

TTF1 is a transcriptional factor expressed in tissues from the thyroid and lung. Immunohistochemical TTF1 staining is commonly used to identify NET of pulmonary origin as it is highly specific (100%) for pulmonary NET with a lower sensibility, ranging from 35% to 53% (62, 63). OPT – orthopedia homeobox (65) is an extremely useful lung NET marker which is positive in 80% of bronchopulmonary carcinoids and shows much higher sensitivity (80.2% sensitivity and 99.4% specificity) compared to TTF1.

Paired-box genes (PAX) encode a family of nine transcription factors (PAX1-9) important for embryogenesis and organogenesis. PAX8 was found to be expressed in 56-74% of pNET (66, 67), However, specificity is hindered by PAX expression in NETs from the duodenum (75%), stomach (10%) (67, 68).

ISL1 is a transcription factor expressed in pancreatic islet cells and has been shown to be expressed in primary GEP-NETs and, less so, in pulmonary NENs: 59-90% pancreatic, 89% duodenum, 0-16% lung, 0-16% ileum, 0% gastric (69–71). Overall, ISL1 should not be considered entirely specific for pNETs, (overall sensitivity - 69-90% and specificity - 78-88%) considering that sensitivity ranges fall to 67-76% in metastatic pNETs (while specificity increases to 89-98%).

Finally, the sensibility and sensitivity of Pancreatic and Duodenal Homeobox 1 (PDX1) and its role in characterization of NETs is discordant. While some studies found a relatively high specificity and sensibility of PDX1 for pNET (72% expression in primary pNET and 100% in metastatic pNET, with a specificity of 92% and 75% respectively) (72), others demonstrated staining of PDX1 in the rectum, stomach, duodenum, appendix (and rarely in the lung and small bowel) and low percentages of expression in pNET (30%) (62, 73).

An important issue with pancreatic markers is that appendiceal/rectal L-cell tumors often express pancreatic markers such as ISL1, PDX1 and PAX8, as shown above. To overcome this potential pitfall, recent studies have shown that special AT-rich sequence binding protein-2 (SATB2), a transcription factor binding protein, may be used as a specific marker for appendiceal/rectal NETs (it is not expressed in pancreatic/duodenal NETs) (74). Lastly, to distinguish rectal and appendiceal L-cell NETs, positivity for prostatic acid phosphatase confirms rectal origin.

### Question 7: How Should These Markers Be Used (Immunopanels to Identify Sites of Origin)?

Various immunopanels have been proposed in the literature to identify site of origin, based on differential use of transcription factors and hormone/amine products (61, 69, 73, 75, 76). An immunohistochemical panel demonstrating TTF1 positivity, negativity for CDX2, ISL1 and PDX1 supports a diagnosis of pulmonary NEN. In this setting calcitonin and CEA expression study can help pathologist to distinguish medullary thyroid carcinoma and pulmonary NEN (61, 69). Conversely, an immunohistochemical panel showing strong and diffuse positive staining for CDX2 and negativity for TTF1, ISL1 and PDX1 favors a midgut origin (usually ileal or appendiceal) (61, 69, 73). An immunohistochemical panel demonstrating TTF1 negativity, negative or weak staining for CDX2, ISL1 and PDX1 positivity suggests a NEN originating from the pancreas or duodenum (61, 72) (the distinction between a pancreatic versus a duodenal NEN is challenging). An immunohistochemical panel demonstrating TTF1 and PDX1 negativity, negative or weak staining for CDX2 and ISL1 positivity suggests a L-cell NEN (61, 70, 72). Despite the use of multiple markers primary tumor detection often remains challenging and requires clinical and radiologic information to reach the final diagnosis.

### Question 8: Are There Other Immunohistochemical Prognostic Markers for NEN Apart From Ki67?

New prognostic immunomarkers, have been recently proposed in NEN. Most of these markers have been principally investigated in pNET and their role in NENs of different sites still remains to be established.

Cytokeratin-19 (CK19) has been shown to be a prognostic factor for NEN even though its prognostic role seems to vary depending on the subtype of pNET. Indeed, CK19 has been identified as a prognostic factor in pNET, excluding insulinomas, with evidence of correlation between CK19 expression and a more aggressive phenotype (77). CK19 has been shown to be an independent prognostic factor (78) with a 5-year survival of all CK19 negative cases of 100%, with a drop to 47% in CK19 positive neoplasms as confirmed by a recent meta-analysis (79).

Insulinoma associated protein 1 (INSM1), a nuclear transcription factor, is a sensitive and well-validated marker for neuroendocrine differentiation (80). Preliminary studies suggest the potential utility of INSM1 as a prognostic factor, as INSM1 expression seems to correlate with more malignant behavior and with greater propensity of metastasis in gastrointestinal NENs (81).

c-KIT, a tyrosine kinase receptor of the platelet derived growth factor subfamily, was found to be a negative independent prognostic marker in pNET with adverse prognosis in c-KIT positive NENs (82).

The prognostic role of DAXX/ATRX expression is more controversial. Some studies have shown loss of expression of DAXX/ATRX to be associated with more aggressive behavior and shorter disease-free survival (83, 84). In contrast, other observations appear to show an improved overall survival in tumors showing loss of DAXX/ATRX (85, 86).

## Conclusions

In conclusion, patient management should be based on multidisciplinary decisions based on precise and specific comprehension of information and communication. Clinicians require an understanding of classification systems (which change over time) and the importance of novel markers which may aid in diagnosis and prognosis as well as concise and standardized pathology reports. In keeping with this last statement, an example of the minimum requirements in pathology datasets is shown in **Table 1** and should be a baseline for all neuroendocrine tumor professionals (87).

**Table 1:** Minimum and optional requirements for a pathology report of gastroenteropancreatic neuroendocrine neoplasm [adapted from Volante et al. (87)].

Minimum Requirements	WHO used (2017-2019) for pathology report
	Differentiation and WHO tumor type (NET, NEC, MINEN), if NEC large or small cell, if MINEN, histotype of NE and non-NE components
	Tumor Grade (<3% for G1, 3-20% G2, > 20% G3) for NET
	Ki-67 index as precise value (%)
	Size and location
	Depth of invasion
	Lympho-vascular invasion (present/absent)
	Perineural invasion (present/absent)
	Lymph node status (number evaluated nodes, number of positive nodes)
	R status and description of margins
	Immunohistochemical markers used for identification of primary, in case of biopsy
	Immunohistochemical markers performed and relative results
	pTNM stage (AJCC/WHO/UICC)
Optional Requirements	Ki-67% on different site (primary and metastases)
	Mitotic index as value (x2 mm^2^)
	If positive lymph node, description of presence/absence of extra nodal extension
	Hormone positivity on immunohistochemistry
	Somatostatin receptor immunohistochemistry (not for routine patology report)

NET, neuroendocrine tumor; NEC, neuroendocrine carcinoma; MINEN, mixed neuroendocrine-non-neuroendocrine neoplasms; AJCC, American Joint Commission on Cancer; WHO, World Health Organization; UICC, Union for International Cancer Control.

## Data Availability Statement

The original contributions presented in the study are included in the article/**Supplementary Material**. Further inquiries can be directed to the corresponding author.

## Author Contributions

MA and FG conceived the study, wrote and finalized the manuscript. GP, FL, and CR contributed to the collection of information and references, writing of the manuscript and approval of the final manuscript. AF and AC supervised and reviewed the manuscript. All authors contributed to the article and approved the submitted version.

## Funding 

This work was supported by the Italian Ministry of Education, University and Research (MIUR): PRIN 2017Z3N3YC.

## Conflict of Interest

The authors declare that the research was conducted in the absence of any commercial or financial relationships that could be construed as a potential conflict of interest.
