# Supplementary material for: Pathology Reporting in Neuroendocrine Neoplasms of the Digestive System: Everything You Always Wanted to Know but Were Too Afraid to Ask
Source: Front Endocrinol (Lausanne). 2021 Apr 23;12:680305. doi: 10.3389/fendo.2021.680305 (PMC8104083; doi:10.3389/fendo.2021.680305)
Supplement: Supplementary file 1 [file DataSheet_1.docx]

| **QUESTIONS** | **SELECTED** | **RATE of VOTES** |
| --- | --- | --- |
| **Questions on Morphology** | |  |
| What morphological features suggest a NET G3 neoplasm? | **Yes (question 1)** | **53%** |
| What Classification system for neuroendocrine neoplasms of the digestive system should I be expecting in a pathology report? | **Yes (question 1)** | **65%** |
| Is it always possible to distinguish small cell neurocarcinoma from large cell neuroendocrine carcinoma? | No |  |
| Is there an adequate number of lymph nodes following nodal dissection for ileal NETs? | No |  |
| Is there an adequate number of lymph nodes following nodal dissection for pancreatic NETs? | No |  |
| What is the clinical impact of mesenteric tumor deposits for small bowel NETs? | No |  |
| What is the clinical impact of angio- and perineural invasion in NETs? | No |  |
| How should I interpret a pathology report showing a digestive system mixed neuroendocrine non-neuroendocrine carcinoma? | **Yes (question 2)** | **61%** |
| **Questions on Grading** | |  |
| How reliable is Ki67 evaluation on cytological samples? | **Yes (question 3)** | **72%** |
| Should all metastatic sites be graded separately? | No |  |
| Does grade change between sites and over time? | **Yes (question 4)** | **55%** |
| How reliable is Ki67 evaluation on biopsy samples? | **Yes (question 3)** | **70%** |
| Is Ki67 mandatory for thoracic NET classification? | No |  |
| Is mitotic count mandatory for GEP-NET classification? | No |  |
| Is there a recommended method for Ki67 evaluation? | No |  |
| **Questions on Immunohistochemistry** | |  |
| Is CgA always necessary to diagnose neuroendocrine neoplasms? | No |  |
| Does immunohistochemical positivity for hormonal markers imply a functional syndrome? | No |  |
| How sensitive/specific are site of origin markers (TTF1, CDX2, PAX8, ISL1, PDX1)? | **Yes (question 6)** | **97%** |
| How should these markers be used (immunopanels to identify sites of origin)? | **Yes (question 7)** | **80%** |
| Is it necessary to evaluate SSRs on neoplastic cells by immunohistochemistry? | **Yes (question 5)** | **55%** |
| Is it always possible to identify origin from metastatic tissue samples? | No |  |
| Are there other IIC prognostic markers for NETs? | **Yes (question 8)** | **77%** |
| What can DAXX/ATRX immuno-evaluation add to pancreatic NET management? | No |  |
| Are there new, interesting, immunomarkers on the horizon in NETs? | No |  |

**Table 1S.** All questions scored during the poll. In bold the 8 top scored questions with rate of votes received. NETs, neuroendocrine tumors; GEP-NETs, gastroenteropancreatic neuroendocrine tumors; CgA, Chromogranin A.
